# Identification of Hydroxyanthraquinones as Novel Inhibitors of Hepatitis C Virus NS3 Helicase

**DOI:** 10.3390/ijms160818439

**Published:** 2015-08-07

**Authors:** Atsushi Furuta, Masayoshi Tsubuki, Miduki Endoh, Tatsuki Miyamoto, Junichi Tanaka, Kazi Abdus Salam, Nobuyoshi Akimitsu, Hidenori Tani, Atsuya Yamashita, Kohji Moriishi, Masamichi Nakakoshi, Yuji Sekiguchi, Satoshi Tsuneda, Naohiro Noda

**Affiliations:** 1 Department of Life Science and Medical Bioscience, Waseda University, 2-2 Wakamatsu-cho, Shinjuku-ku, Tokyo 162-8480, Japan; E-Mails: atsushi.5961@ruri.waseda.jp (A.F.); tatuki-miyamoto@asagi.waseda.jp (T.M.); 2 Biomedical Research Institute, National Institute of Advanced Industrial Science and Technology (AIST), 1-1-1 Higashi, Tsukuba, Ibaraki 305-8566, Japan; E-Mail: y.sekiguchi@aist.go.jp; 3 Institute of Medical Chemistry, Hoshi University, 2-4-41 Ebara, Shinagawa-ku, Tokyo 142-8501, Japan; E-Mails: tsubuki@hoshi.ac.jp (M.T.); hyonba@yahoo.co.jp (M.E.); 4 Department of Chemistry, Biology and Marine Science, University of the Ryukyus, Nishihara, Okinawa 903-0213, Japan; E-Mail: jtanaka@sci.u-ryukyu.ac.jp; 5 Radioisotope Center, The University of Tokyo, 2-11-16 Yayoi, Bunkyo-ku, Tokyo 113-0032, Japan; E-Mails: salam_bio26@yahoo.com (K.A.S.); akimitsu@ric.u-tokyo.ac.jp (N.A.); 6 Environmental Measurement Research Institute, National Institute of Advanced Industrial Science and Technology (AIST), 16-1 Onogawa, Tsukuba, Ibaraki 305-8569, Japan; E-Mail: h.tani@aist.go.jp; 7 Department of Microbiology, Division of Medicine, Graduate School of Medicine and Engineering, University of Yamanashi, 1110 Shimokato, Chuo-shi, Yamanashi 409-3898, Japan; E-Mails: atsuyay@yamanashi.ac.jp (A.Y.); kmoriishi@yamanashi.ac.jp (K.M.); 8 Department of Pharmaceutical Sciences, Toho University, 2-2-1 Miyama, Funabashi-shi, Chiba 274-8510, Japan; E-Mail: nakakoshi@phar.toho-u.ac.jp

**Keywords:** hepatitis C virus, NS3 helicase, fluorescence resonance energy transfer, inhibitor, hydroxyanthraquinone, hypericin, sennidin A

## Abstract

Hepatitis C virus (HCV) is an important etiological agent of severe liver diseases, including cirrhosis and hepatocellular carcinoma. The HCV genome encodes nonstructural protein 3 (NS3) helicase, which is a potential anti-HCV drug target because its enzymatic activity is essential for viral replication. Some anthracyclines are known to be NS3 helicase inhibitors and have a hydroxyanthraquinone moiety in their structures; mitoxantrone, a hydroxyanthraquinone analogue, is also known to inhibit NS3 helicase. Therefore, we hypothesized that the hydroxyanthraquinone moiety alone could also inhibit NS3 helicase. Here, we performed a structure–activity relationship study on a series of hydroxyanthraquinones by using a fluorescence-based helicase assay. Hydroxyanthraquinones inhibited NS3 helicase with IC_50_ values in the micromolar range. The inhibitory activity varied depending on the number and position of the phenolic hydroxyl groups, and among different hydroxyanthraquinones examined, 1,4,5,8-tetrahydroxyanthraquinone strongly inhibited NS3 helicase with an IC_50_ value of 6 µM. Furthermore, hypericin and sennidin A, which both have two hydroxyanthraquinone-like moieties, were found to exert even stronger inhibition with IC_50_ values of 3 and 0.8 µM, respectively. These results indicate that the hydroxyanthraquinone moiety can inhibit NS3 helicase and suggest that several key chemical structures are important for the inhibition.

## 1. Introduction

Chronic infection with hepatitis C virus (HCV) can result in severe liver diseases, including cirrhosis and hepatocellular carcinoma [1]. The previous standard anti-HCV therapy was a combination of pegylated interferon (PEG-IFN) and ribavirin [2]. However, this combination therapy exerts severe adverse effects and its efficacy decreases for certain HCV genotypes, especially HCV genotype 1 [3,4]. In order to overcome these disadvantages, recent research on anti-HCV drugs has mainly focused on direct-acting antivirals that target viral or host proteins involved in HCV replication [5].

The first major advance pertaining to direct-acting antivirals was the development of NS3 serine protease inhibitors that, when used in combination with PEG-IFN and ribavirin, provide a higher viral clearance rate for HCV genotype 1 infections than the PEG-IFN plus ribavirin combination [6,7,8]. A novel combination therapy involving NS5A and NS5B polymerase inhibitors has been approved, which provides a higher viral clearance rate than the combination of NS3 serine protease inhibitor, PEG-IFN, and ribavirin even without PEG-IFN and ribavirin [9,10,11,12]. However, it is still deemed necessary to further develop novel direct-acting antivirals because of the following factors: adverse effects, such as nausea, anemia, and headache [9]; excessively high treatment cost; and possibility of emergence of drug-resistant HCV mutations and drug–drug interactions [13].

HCV belongs to the *Flaviviridae* family of positive-stranded RNA viruses [14]. A polyprotein expressed from a single open reading frame becomes mature through viral and host-cellular protease processing, leading to the production of structural and nonstructural proteins [5,15]. The NS3 protein is a nonstructural protein that exerts multiple enzymatic functions via serine protease and NTPase/helicase (NS3 helicase) domains at the *N*- and *C*-terminus, respectively [16]. NS3 helicase can unwind various types of double-stranded (ds) nucleic acids, such as dsRNA, dsDNA, and DNA/RNA heteroduplexes, in the 3ʹ–5ʹ direction by hydrolyzing nucleoside triphosphates [17,18,19,20]. Since NS3 helicase is responsible for HCV replication *in vitro* [21] and *in vivo* [22], an inhibitor of NS3 helicase is deemed a potential anti-HCV agent [23]. However, no NS3 helicase inhibitors have entered clinical trials, mainly due to their low efficacy and severe cytotoxicity.

Some anthracyclines, such as doxorubicin, daunomycin, epirubicin, and nogalamycin, as well as their derivatives, have been identified as NS3 helicase inhibitors [24,25]. Anthracyclines have a hydroxyanthraquinone moiety in their chemical structure, and mitoxantrone, which is also known to inhibit NS3 helicase, is an analogue of hydroxyanthraquinone [24]. These findings led us to hypothesize that hydroxyanthraquinone alone could inhibit NS3 helicase.

Here, we performed a structure–activity relationship study on a series of hydroxyanthraquinones by using a fluorescence helicase assay based on fluorescence resonance energy transfer (FRET) that we had developed previously [26,27], with modifications in the fluorescent dyes used, to demonstrate NS3 helicase inhibition by hydroxyanthraquinones and identify several key structures important for inhibition.

## 2. Results and Discussion

### 2.1. Structure–Activity Relationship Study on Hydroxyanthraquinones

A fluorescence helicase assay based on FRET [26,27], with modifications in the fluorescent dyes, was used to examine NS3 helicase inhibition by different compounds. Since hydroxyanthraquinone is known to exhibit a wide range of absorption wavelengths in aqueous solution, ranging from shorter to longer wavelengths (e.g., ~200 up to 700 nm) [28], we used a dsRNA substrate prepared by annealing the 5′ Alexa Fluor 700 (maximum excitation/emission = 702/723 nm)-labeled fluorescence strand to the 3′ Black Hole Quencher (BHQ)-3-labeled quencher strand with the same RNA sequences, as described in previous reports [27,29], to avoid interference due to hydroxyanthraquinone absorption. The concentration of the capture strand was optimized to 400 nM based on the *Z*′ value [30]; the *Z*′ value validates assay conditions as having reliability and reproducibility when its value is close to 1.

**Figure 1:**
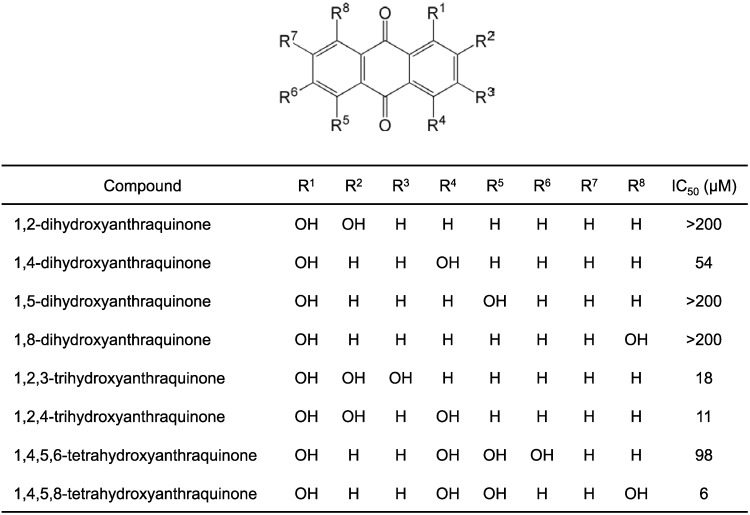
Structures of hydroxyanthraquinones and their IC_50_ values for NS3 helicase inhibition. The IC_50_ values were obtained from inhibition curves (Figure S1). The data in the inhibition curves are presented as mean ± standard deviation (SD) of three replicates using the fluorescence helicase assay. The NS3 helicase activities of samples containing inhibitor were calculated relative to control samples, containing DMSO vehicle instead of inhibitor.

To prove our hypothesis that a hydroxyanthraquinone moiety alone could inhibit NS3 helicase, we first examined 1,4-dihydroxyanthraquinone, which differs from mitoxantrone, a hydroxyanthraquinone analogue with an IC_50_ value of 6.7 µM for NS3 helicase inhibition [24], only in the fact that it lacks two {2-[(2-hydroxyethyl)amino]ethyl}amino groups. As expected, 1,4-dihydroxyanthraquinone inhibited NS3 helicase; the IC_50_ value was 54 µM (Figure 1).

Next, regioisomers of 1,4-dihydroxyanthraquinone, 1,2-dihydroxyanthraquinone, 1,5-dihydroxyanthraquinone, and 1,8-dihydroxyanthraquinone, were examined for NS3 helicase inhibition (Figure 1). The IC_50_ values for 1,2-dihydroxyanthraquinone, 1,5-dihydroxyanthraquinone, and 1,8-dihydroxyanthraquinone were all >200 µM (Figure 1), indicating that these compounds exhibited much less inhibitory activity than 1,4-dihydroxyanthraquinone. This result suggests that the inhibitory activity depends on the number and position of phenolic hydroxyl groups. The phenolic hydroxyl groups at positions 1, 4, 5, and 8 in the anthraquinone structure are known to be responsible for the “keto-phenol system”, which consists of the tautomeric anthraquinoid structures between the ketone and phenolic hydroxyl groups [28,31,32]. While there is only one keto-phenol system in 1,2-dihydroxyanthraquinone, there are two keto-phenol systems in 1,5-dihydroxyanthraquinone. In the structure of 1,8-dihydroxyanthraquinone, one ketone group is shared in two keto-phenol systems. Since 1,4-dihydroxyanthraquinone differs from the above three regioisomers in terms of having two keto-phenol systems positioned on the same benzene ring, we considered that this characteristic structure could be key for NS3 helicase inhibition.

Two trihydroxyanthraquinones, 1,2,3-trihydroxyanthraquinone and 1,2,4-trihydroxyanthraquinone, were also examined for NS3 helicase inhibition. The IC_50_ values for 1,2,3-trihydroxyanthraquinone and 1,2,4-trihydroxyanthraquinone were 18 and 11 µM, respectively (Figure 1), which indicates that these compounds exhibited stronger inhibitory activity than 1,4-dihydroxyanthraquinone. Since 1,2,3-trihydroxyanthraquinone and 1,2,4-trihydroxyanthraquinone exhibited similar inhibitory activity, it can be suggested that the three phenolic hydroxyl groups positioned on the same benzene ring, one of which forms a keto-phenol system, have inhibitory activity similar to that of two keto-phenol systems positioned on the same benzene ring with one phenolic hydroxyl group. The results also suggest that the addition of phenolic hydroxyl groups to the benzene ring with two keto-phenol systems would further increase the inhibitory activity compared with that seen for 1,4-dihydroxyanthraquinone.

Two tetrahydroxyanthraquinones, 1,4,5,6-tetrahydroxyanthraquinone and 1,4,5,8-tetrahydroxyanthraquinone, were also examined for NS3 helicase inhibition. The IC_50_ values for 1,4,5,6-tetrahydroxyanthraquinone and 1,4,5,8-tetrahydroxyanthraquinone were 98 and 6 µM, respectively (Figure 1), indicating that 1,4,5,8-tetrahydroxyanthraquinone inhibited NS3 helicase even more strongly than 1,2,4-trihydroxyanthraquinone. While 1,4,5,8-tetrahydroxyanthraquinone has four keto-phenol systems, in which each pair shares a ketone group, 1,4,5,6-tetrahydroxyanthraquinone has three keto-phenol systems, two of which share a ketone group. This result suggests that increasing the number of pairs in keto-phenol systems positioned on the same benzene ring would further increase the inhibitory activity.

While many hydroxyanthraquinones are naturally occurring compounds and have already been commercially used (e.g., for dyes), these compounds also have various biological activities, such as immunosuppressive activity [33], mutagenic activity [34], genotoxic activity [35], carcinogenic activity [36,37,38], toxic and tumorigenic activities [39], intestinal motility inhibitory activity [40], and osteosarcoma and antitumor activity [41]. To our knowledge, this study is the first to identify hydroxyanthraquinones as HCV NS3 helicase inhibitors for use as potential antiviral agents.

### 2.2. Inhibitory Activity of Hypericin and Sennidin A

**Figure 2:**
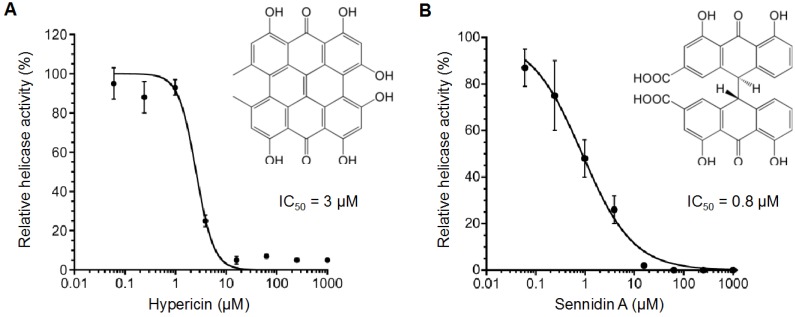
Inhibition curves of (**A**) hypericin and (**B**) sennidin A generated using the fluorescence helicase assay. The NS3 helicase activities of samples containing inhibitor were calculated relative to control samples containing DMSO vehicle instead of inhibitor. The data are presented as mean ± standard deviation of three replicates.

Aurintricarboxylic acid has a keto-phenol system like the hydroxyanthraquinones and is known to inhibit NS3 helicase in its multimerized forms in aqueous solutions [42]. Therefore, we hypothesized that the multimerization of hydroxyanthraquinones might also affect their NS3 helicase inhibition. We therefore examined the inhibitory activity of hypericin and sennidin A, which both have two hydroxyanthraquinone-like moieties, *i.e.*, a doubled anthracene-skeleton structure with four keto-phenol systems, with each pair sharing one ketone group. Hypericin and sennidin A inhibited NS3 helicase even more efficiently than 1,4,5,8-tetrahydroxyanthraquinone, with IC_50_ values of 3 and 0.8 µM, respectively (Figure 2A,B). This inhibition was further confirmed with a gel-based helicase assay, where the NS3 helicase inhibition observed was quite similar to that in the fluorescence helicase assay (Figure S2).

Hypericin is one of the major active constituents of the plant *Hypericum*. It can generate superoxide anions and has a high quantum yield of singlet oxygen, which results in a wide variety of bioactivities, such as antitumor, antiviral, and antidepressant effects [43].

Sennidin A is known to stimulate glucose incorporation in rat adipocytes [44] and has been reported as being useful in tumor necrosis therapy owing to its avidity for necrotic tumors [45].

To our knowledge, this study is the first to identify hypericin and sennidin A as HCV NS3 helicase inhibitors for use as potential antiviral agents. In addition, our findings suggest that multimerization of hydroxyanthraquinones would further increase their inhibitory activity.

### 2.3. Effect of Hypericin and Sennidin A on HCV Replication and Cytotoxicity

We examined the effect of hypericin and sennidin A on HCV replication by using the HCV subgenomic replicon system (Table 1). Both the compounds suppressed HCV replication in a dose-dependent manner; however, anti-HCV activity of sennidin A was rather small (EC_50_ > 80 µM) compared with its IC_50_ value of 0.8 µM for NS3 RNA unwinding activity (Figure 2B), and the CC_50_ value for cytotoxicity of sennidin A was >80 µM. These results suggest that the compound was not well delivered into cytoplasm of Huh-7 cell line presumably due to its low membrane permeability caused by the two negatively-charged carboxyl groups within the molecule in aqueous solution. In contrast to the above result, hypericin well suppressed HCV replication (EC_50_ = 3.5 ± 0.2 µM; CC_50_ = 41.1 ± 9.5 µM). These results and Figure 2A indicate that the inhibition of HCV replication and NS3 helicase activity occur at a similar inhibitor concentration for hypericin, suggesting that the anti-HCV activity of the compound is associated with the inhibition of NS3 helicase. In addition, the selectivity index (SI) of hypericin can be calculated as 11.7 (CC_50_/EC_50_), indicating that the compound has relatively high specificity for HCV compared with the inhibitors previously reported [46]. Furthermore, the results suggest that hydroxyanthraquinones may have potential anti-HCV activity.

**Table 1:** Effect of hypericin and sennidin A on HCV replication and cytotoxicity.

Compound	EC_50_ (µM)	CC_50_ (µM)	SI
Hypericin	3.5 ± 0.2	41.1 ± 9.5	11.7
Sennidin A	>80	>80	n.d.

EC_50_, fifty percent effective concentration based on the inhibition of HCV replication; CC_50_, fifty percent cytotoxicity concentration based on the reduction in cell viability; SI, selectivity index (CC_50_/EC_50_); n.d., not determined. The EC_50_ and CC_50_ values are presented as mean ± standard deviation of three replicates.

### 2.4. Effect of Hypericin on NS3 ATPase Activity

**Figure 3:**
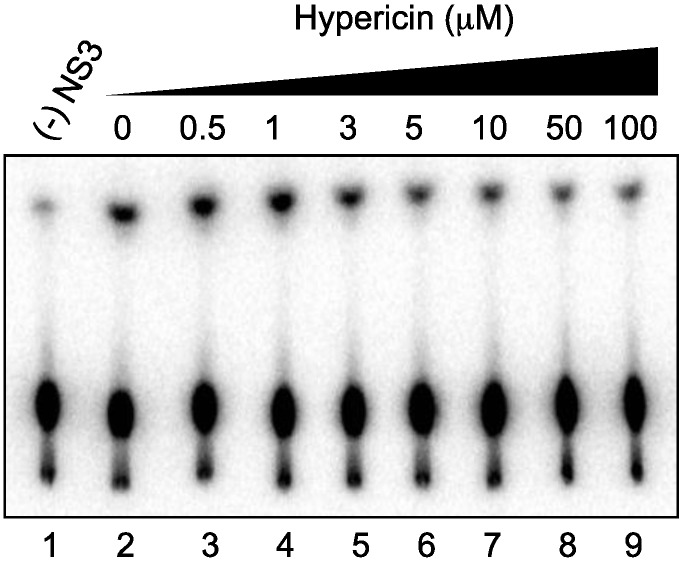
Effect of hypericin on NS3 ATPase activity. Activity was demonstrated by autoradiography of an ATPase assay using [γ-^32^P]ATP. Lane 1 contains the control reaction without NS3; Lanes 2–9 show the ATP hydrolysis reaction with poly(U) RNA at increasing concentrations (0–100 μM) of hypericin.

As the unwinding ability of NS3 helicase is dependent on ATP hydrolysis, the amount of inorganic phosphate (Pi) released from radioisotope-labeled ATP ([γ-^32^P]ATP) was measured to determine the effects of hypericin on the ATPase activity of NS3 (Figure 3). The released ^32^Pi was separated by thin-layer chromatography and visualized using autoradiography. The density of the upper spots corresponding to Pi, which represents ATPase activity, decreased dose-dependently, particularly in the concentration range of 1–5 µM hypericin. This result indicates that hypericin inhibits NS3 ATPase activity in a concentration range similar to that in which RNA unwinding is inhibited (Figure 2A). It is thus likely that hypericin inhibits NS3 helicase via the inhibition of ATPase activity.

### 2.5. Effect of Hypericin on NS3 RNA-Binding Activity

**Figure 4:**
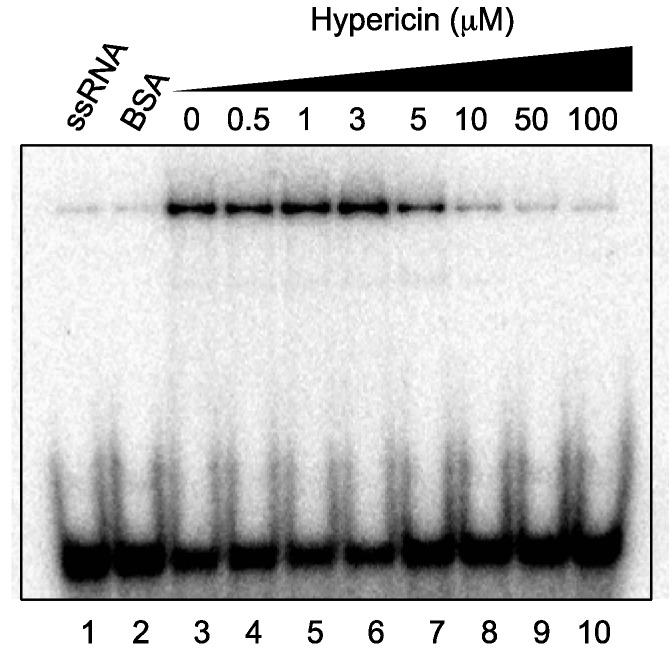
Effect of hypericin on NS3 RNA-binding activity. Activity was assessed by autoradiography of a gel mobility-shift assay using ^32^P-labeled ssRNA. Lanes 1 and 2 contain control reactions with heat-denatured ssRNA and 300 nM BSA (instead of NS3), respectively; Lanes 3–10 show the RNA-binding reaction with increasing concentrations (0–100 μM) of hypericin.

As RNA binding is required for NS3 helicase activity, the effect of hypericin on NS3 RNA-binding activity was examined by gel mobility-shift assay (Figure 4). As a control, the non-specific binding of ssRNA to bovine serum albumin (BSA) was assessed (lane 2). The density of the upper bands corresponding to the NS3–ssRNA complex, which represents NS3 RNA-binding activity, decreased dose-dependently in the presence of hypericin, particularly in the concentration range of 3–10 µM. The data presented in Figure 2A and Figure 4 reveal that the NS3 helicase and RNA-binding activities decreased at a similar inhibitor concentration for hypericin, suggesting that the inhibition of NS3 helicase by the compound is associated with inhibition of RNA-binding activity.

The fact that hypericin inhibits NS3 ATPase and RNA-binding activities may be reasonable, since aurintricarboxylic acid, which has a keto-phenol system and inhibits NS3 helicase in its multimerized form in aqueous solutions, has already been reported to inhibit both these activities [42]. As aurintricarboxylic acid has been suggested to prevent NS3 helicase from interacting with either ATP or nucleic acids by causing a conformational change, such a model might also explain the inhibitory mechanism of hypericin. Furthermore, our findings suggest that hydroxyanthraquinones may similarly inhibit NS3 ATPase and RNA-binding activities.

## 3. Experimental Section

### 3.1. Chemicals

All the compounds used in this study, except for 1,4,5,8-tetrahydroxyanthraquinone, were purchased commercially: 1,2-dihydroxyanthraquinone (Wako, Osaka, Japan), 1,4-dihydroxyanthraquinone (Tokyo Chemical Industry, Tokyo, Japan), 1,5-dihydroxyanthraquinone (Tokyo Chemical Industry), 1,8-dihydroxyanthraquinone (Wako), 1,2,3-trihydroxyanthraquinone (Alfa Aesar, Ward Hill, MA, USA), 1,2,4-trihydroxyanthraquinone (Sigma-Aldrich, St. Louis, MO, USA), 1,4,5,6-tetrahydroxyanthraquinone (Sigma-Aldrich), hypericin (Wako), and sennidin A (ChromaDex, Irvine, CA, USA). We synthesized 1,4,5,8-tetrahydroxyanthraquinone as described previously [47].

### 3.2. FRET-Based Fluorescence Helicase Assay

The FRET-based fluorescence helicase assay was performed as described previously [27,29], with modifications in the fluorescent dyes used. The dsRNA substrate was prepared by annealing the 5′ Alexa Fluor 700-labeled fluorescence strand to the 3′ BHQ3-labeled quencher strand in a 1:2 molar ratio. The dsRNA substrate contained the 3′-overhang that is necessary for the NS3 helicase to bind to it prior to duplex unwinding. The capture strand had sequence complementary to the quencher strand and prevented the unwound duplexes from reannealing. All the nucleic acid strands had the same nucleic acid sequences as described previously [27,29] and were purchased from Japan Bio Services (Saitama, Japan). The reaction mixture contained 25 mM MOPS-NaOH (pH 6.5), 3 mM MgCl_2_, 2 mM dithiothreitol, 4 U RNasin (Promega, Madison, WI, USA), 50 nM dsRNA substrate, 400 nM capture strand, 5 mM ATP, a serial dilution of test compounds in DMSO vehicle, and 240 nM NS3 helicase in a total reaction volume of 20 µL. The full-length HCV NS3 protein, with serine protease and NTPase/helicase activities, was expressed and purified as described previously [27,48].

The reaction was started by the addition of HCV NS3 helicase and was performed at 37 °C for 30 min in a SpectraMax Gemini XS microplate reader (Molecular Devices, Sunnyvale, CA, USA). The fluorescence intensity was recorded every 20 s for 30 min. Helicase activity was calculated as the initial reaction velocity relative to that of the control (in the absence of a test compound but presence of DMSO vehicle). The IC_50_ was calculated using KaleidaGraph (Synergy Software, Reading, PA, USA) by fitting plots of % activity *vs.* [I] using Equation (1) unless otherwise stated [49]:

(1) $$\%\text{ Activity}=\;\frac{100}{1+{\left(\left[I\right]/{\text{IC}}_{50}\right)}^{h}}$$

where *h* is the Hill coefficient, and [*I*] is the inhibitor concentration.

### 3.3. Gel-Based Helicase Assay

A gel-based helicase assay was performed as described previously [29]. The dsRNA substrate was prepared by annealing the 5′ Alexa Fluor 488-labeled fluorescence strand to the non-labeled complementary strand in a 1:2 molar ratio. The dsRNA substrate and the capture strand had the same nucleic acid sequences as those used in the FRET-based fluorescence helicase assay, and were purchased from Japan Bio Services. The reaction mixture for HCV NS3 helicase had the same components as those used in the FRET-based fluorescence helicase assay, with increasing concentrations of a test compound in a total reaction volume of 20 µL, except for the fact that the concentration of the capture strand was 100 nM. The reaction was started by the addition of HCV NS3 helicase and performed at 37 °C for 60 min using the GeneAmp PCR System 2700 (Applied Biosystems, Foster City, CA, USA). The reaction was stopped by the addition of 5 µL of helicase termination buffer, containing 10 mM Tris-HCl (pH 7.5), 50 mM EDTA, 30% glycerol, 0.06% bromophenol blue, and 0.12% Orange G. The inhibition of NS3 helicase was analyzed using a native 20% polyacrylamide–Tris/borate/EDTA (TBE) gel, and labeled RNAs were visualized using a Typhoon 9210 scanner (GE Healthcare, Waukesha, WI, USA). Cholesterol sulfate (IC_50_ = 1.7 µM) [50] from Avanti Polar Lipids (Alabaster, AL, USA) was used at a final concentration of 100 µM as a positive control for NS3 helicase inhibition. The helicase activity was calculated as the ratio of the signal intensity derived from ssRNA in the sample containing inhibitor to that in the control sample containing DMSO vehicle instead of inhibitor.

### 3.4. HCV Replicon Assay

The Huh-7 cell line harboring the subgenomic replicon RNAs of HCV genotype 1b strain N [51] was seeded at 2 × 10^4^ cells per well in a 48-well plate and incubated at 37 °C for 24 h. The cells were treated with hypericin or sennidin A at various concentrations at 37 °C for 72 h, and then lysed in cell culture lysis reagent (Promega). A luciferase assay system (Promega) was used to determine the luciferase activity, and the luminescence was measured using a Luminescencer-JNR AB-2100 (ATTO, Tokyo, Japan), corresponding to the expression level of the HCV replicon. The anti-HCV activity of hypericin or sennidin A was calculated as the ratio of the intensity of the luminescence in the sample containing inhibitor to that in the control sample containing DMSO vehicle instead of inhibitor.

### 3.5. Cytotoxicity Assay

The Huh-7 cell line harboring the subgenomic replicon RNAs of HCV genotype 1b strain N [51] was seeded at 2 × 10^4^ cells per well in a 48-well plate and incubated at 37 °C for 24 h. The cells were treated with hypericin or sennidin A at various concentrations at 37 °C for 72 h. The MTS assay was carried out to determine cytotoxicity using a CellTiter 96 aqueous one-solution cell proliferation assay kit (Promega) according to the manufacturer’s instructions. The cytotoxicity of hypericin or sennidin A was calculated as the ratio of the intensity of the luminescence in the sample containing inhibitor to that in the control sample containing DMSO vehicle instead of inhibitor.

### 3.6. ATPase Assay

NS3 ATPase activity was determined directly by monitoring [γ-^32^P]ATP hydrolysis by thin-layer chromatography, as described previously [27,29]. The reaction mixture contained 25 mM MOPS-NaOH (pH 7.0), 1 mM dithiothreitol, 5 mM MgCl_2_, 5 mM CaCl_2_, 1 mM [γ-^32^P]ATP (Muromachi Yakuhin, Tokyo, Japan), 300 nM NS3, 0.1 μg/μL poly(U) ssRNA (Sigma-Aldrich), and increasing concentrations of hypericin in a total reaction volume of 10 μL. The reaction was conducted at 37 °C for 10 min, and stopped by the addition of 10 mM EDTA. Two microliters of each reaction mixture were then spotted onto a polyethyleneimine cellulose sheet (Merck, Darmstadt, Germany) and developed in 0.75 M LiCl/1 M formic acid solution for 20 min. The cellulose sheet was dried, and the released [γ-^32^P]phosphoric acid was visualized using an Image Reader FLA-9000 (Fuji film, Tokyo, Japan).

### 3.7. RNA-Binding Assay

NS3 RNA-binding activity was determined by gel mobility-shift assay, as described previously [27,29]. The ssRNA (5′-UGAGGUAGUAGGUUGUAUAGU-3′) synthesized by Gene Design (Osaka, Japan) was labeled at the 5′-end with [γ-^32^P]ATP (Muromachi Yakuhin) using T4 polynucleotide kinase (Toyobo, Osaka, Japan) at 37 °C for 60 min, and purified using the phenol-chloroform extraction method. The reaction mixture contained 30 mM Tris-HCl (pH 7.5), 100 mM NaCl, 2 mM MgCl_2_, 1 mM dithiothreitol, 20 U RNasin Plus (Promega), 300 nM NS3, 0.5 nM ^32^P-labeled ssRNA, and increasing concentrations of hypericin in a total reaction volume of 20 μL. The reaction was performed at room temperature for 15 min. An equal volume of a dye solution containing 0.025% bromophenol blue and 10% glycerol in 0.5× TBE was then added to each reaction mixture, and samples were separated in a native 6% polyacrylamide gel. The labeled RNA bands were visualized using an Image Reader FLA-9000 (Fujifilm, Tokyo, Japan).

## 4. Conclusions

In this study, we performed structure–activity relationship analyses on a series of hydroxyanthraquinones by using a fluorescence-based helicase assay. Hydroxyanthraquinones inhibited NS3 helicase with IC_50_ values in the micromolar range, which varied depending on the number and position of phenolic hydroxyl groups. In particular, 1,4,5,8-tetrahydroxyanthraquinone was found to strongly inhibit NS3 helicase with an IC_50_ value of 6 µM. This result indicates that a hydroxyanthraquinone moiety alone can inhibit NS3 helicase and suggests that two keto-phenol systems positioned on the same benzene ring constitute a key structure for NS3 helicase inhibition. Furthermore, the data as a whole suggest that increasing the number of pairs of keto-phenol systems positioned on the same benzene ring further increases the inhibitory activity.

Hypericin and sennidin A, both of which have two hydroxyanthraquinone-like moieties, *i.e.*, the doubled anthracene-skeleton structure with four keto-phenol systems in which each pair shares one ketone group, exerted even stronger inhibition than 1,4,5,8-tetrahydroxyanthraquinone, with IC_50_ values of 3 and 0.8 µM, respectively. These results suggest that multimerization of hydroxyanthraquinones increases their inhibitory activity.

Furthermore, although anti-HCV activity of sennidin A was rather small (EC_50_ > 80 µM) compared with its IC_50_ value for NS3 RNA unwinding activity, hypericin well suppressed HCV replication (EC_50_ = 3.5 ± 0.2 µM) at concentrations similar to that at which RNA unwinding was inhibited, with the CC_50_ value of 41.1 ± 9.5 µM. The result suggests that the anti-HCV activity of hypericin is associated with the inhibition of NS3 helicase and the hydroxyanthraquinones have potential anti-HCV activities. In addition, hypericin inhibited NS3 ATPase and RNA-binding activities at concentrations similar to that at which RNA unwinding was inhibited, suggesting that the inhibition of NS3 helicase is associated with inhibition of NS3 ATPase and RNA-binding activities and that hydroxyanthraquinones may similarly inhibit these activities.

We believe that our findings will prove useful in the further development of novel NS3 helicase inhibitors.

## Supplementary Materials

Supplementary materials can be found at http://www.mdpi.com/1422-0067/16/08/18439/s1.
