# Supplementary material for: Identification of Hydroxyanthraquinones as Novel Inhibitors of Hepatitis C Virus NS3 Helicase
Source: Int J Mol Sci. 2015 Aug 7;16(8):18439–53. doi: 10.3390/ijms160818439 (PMC4581254; doi:10.3390/ijms160818439)
Supplement: Supplementary file 1 [file ijms-16-18439-s001.pdf]

Supplementary Information

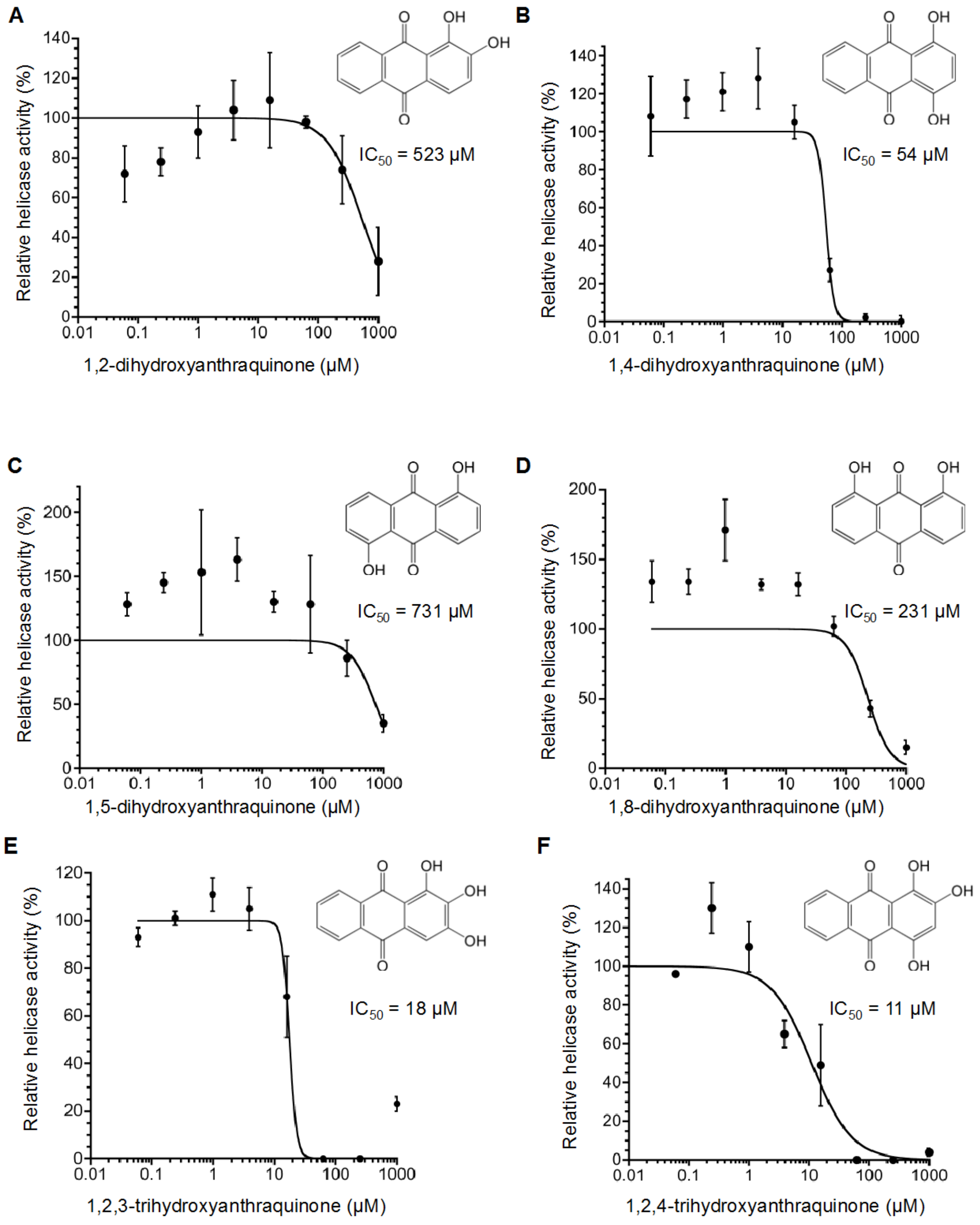

Figure S1. *Cont.*

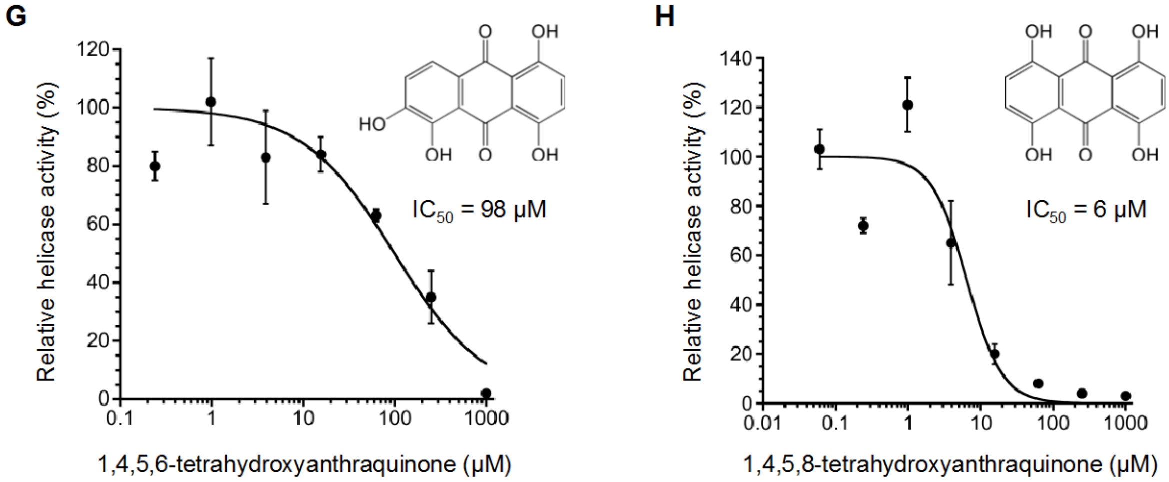

Figure S1. Inhibition curves of hydroxyanthraquinones (**A**–**H**) generated using a fluorescence helicase assay. NS3 helicase activities of samples containing inhibitor were calculated relative to those of control samples containing DMSO vehicle rather than inhibitor. The data are presented as mean ± standard deviation of three replicates.

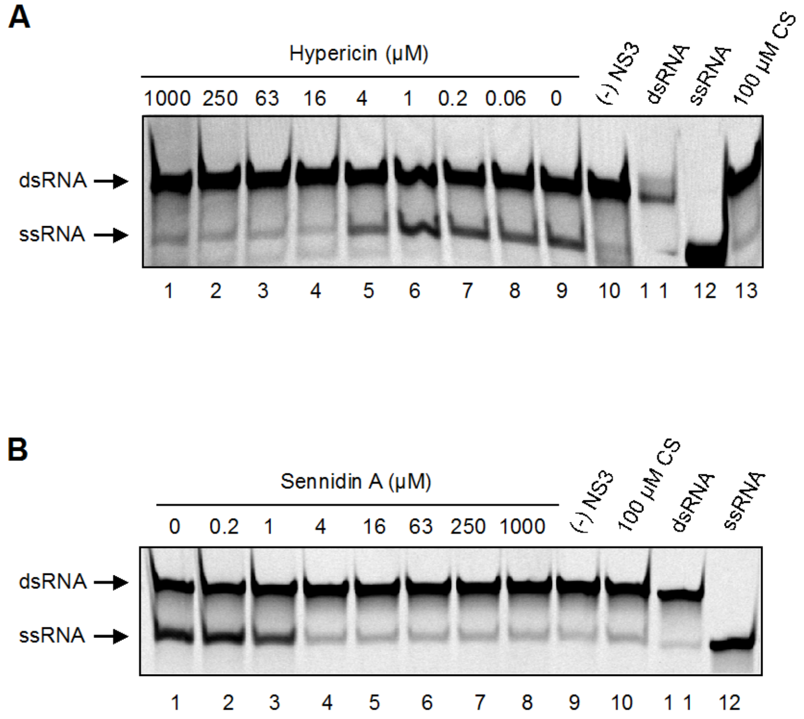

Figure S2. Gel images representing the inhibitory effects of hypericin (**A**) and sennidin A (**B**) in a gel-based helicase assay. The dsRNA was incubated with NS3 in the presence of increasing concentrations of inhibitor, as indicated above the gel images. Lane 10 in **A** and lane 9 in **B** show the control reaction in the absence of NS3. Cholesterol sulfate (CS) (IC_{50} = 1.7 μM) was used at a final concentration of 100 μM as positive control for NS3 helicase inhibition, as shown in lane 13 in **A** and lane 10 in **B**. Fluorescence-labeled dsRNA and ssRNA were applied to lanes 11 and 12, respectively, in **A** and **B**.
